# Inter‐rater reliability and clinical utility of the BASED score in infantile epileptic spasms syndrome

**DOI:** 10.1002/epd2.70255

**Published:** 2026-04-21

**Authors:** T. P. Cramer, B. Johnsen, V. S. Hansen, E. Qerama, T. S. Engedal

**Affiliations:** ^1^ Department of Clinical Neurophysiology Aarhus University Hospital Aarhus Denmark; ^2^ Department of Clinical Medicine Aarhus University Aarhus Denmark

**Keywords:** BASED score, EEG, epileptic encephalopathy, hypsarrhythmia, infantile spasms, west syndrome

## Abstract

**Objective:**

Hypsarrhythmia is the classical EEG pattern of children with infantile epileptic spasms syndrome (IESS). Multifocal spikes, slow waves of large amplitude, and chaoticity are its main characteristics, but these lack clear definitions, and the interrater reliability (IRR) is poor. The aim of this study was to substantiate the use of the Burden of Amplitudes and Epileptiform Discharges (BASED) score for children with hypsarrhythmia on initial EEG and to compare the IRR to traditional scoring.

**Methods:**

We identified before and after treatment EEGs from 20 consecutive patients from a local database. Clinical remission was defined as the remission of spasms. Three experienced Danish neurophysiologists, blinded to clinical information, rated each EEG separately using traditional visual analysis and the BASED score. A BASED score ≥4 and any hypsarrhythmic pattern were considered consistent with an epileptic encephalopathy. IRR was estimated using Gwet's AC1.

**Results:**

IRR for scoring an epileptic encephalopathy was significantly better when using the BASED score compared with the traditional method (0.88 vs. 0.62, *p* = 0.01). Complete agreement was observed in 36 out of 40 EEGs using the BASED score vs. 28 out of 40 using the traditional method (Fisher's exact test: *p* = 0.048). IRR was near perfect or substantial in all but one of the individual components of the BASED score. Clinical remission was seen in 14 out of 19 patients, and the clinical course was consistent with EEG in 18 out of 19 patients using the BASED score and in 16 out of 19 patients using the traditional method (Fisher's exact test: *p* = 0.60).

**Significance:**

The BASED score demonstrated very high interrater reliability, exceeding that of traditional hypsarrhythmia scoring. It also corresponded closely with the clinical course, supporting its validity.

**Abbreviations**
BASED: burden of amplitudes and epileptiform discharges
GMFS: grouped multifocal spikes
HSS: hypsarrhythmia scoring system
IESS: infantile epileptic spasms syndrome
IRR: interrater reliability

**Key points**

- The original definition of hypsarrhythmia was imprecise.
- Attempts to score infantile epileptic spasms syndrome were never validated.
- Early recognition of infantile epileptic spasms syndrome may improve outcome.
- In this study, the BASED score demonstrated very high interrater reliability.
- The BASED score corresponded to the clinical outcome in 18 of 19 patients.

## 1 INTRODUCTION

Infantile epileptic spasms syndrome (IESS) is a severe neurological condition that presents in 1.5–4.5 per 10.000 infants.^1^, ^2^, ^3^ It shows no racial predilection but has a slightly higher incidence in males.^1^, ^4^ By definition the age of onset is between 1 and 24 months, but most cases present between 3 and 12 months after birth.^1^ Spasms, the hallmark clinical feature of IESS, are a recognizable clinical characteristic, which typically last less than 2 s and involve especially the axial musculature.^5^, ^6^ Spasms typically appear in clusters, increasing in frequency and/or prominence over a series of minutes, after which they decrease in a crescendo‐decrescendo pattern.^7^, ^8^ On EEG about 60% of patients with IESS will present with an interictal pattern known as hypsarrhythmia,^9^, ^10^ which is classically characterized by multifocal spikes, slow waves of large amplitude, and chaoticity.^1^, ^7^ Hypsarrhythmia, epileptic spasms, and developmental delay are the three criteria for West syndrome, the progenitor term to IESS.^11^

The overall prognosis of children with IESS is poor even with proper treatment, with a mortality rate of 5%–30%, while only around 33% of patients achieve long‐term freedom from seizures, and approximately 25% achieve an IQ above 68.^12^, ^13^, ^14^ Early recognition might improve outcome but is complicated by several factors. Epileptic spasms are easily overlooked or misinterpreted as a benign phenomenon, as they can present so mildly that even healthcare professionals have difficulties acknowledging them without concurrent EEG monitoring.^1^, ^5^ Even when spasms are recognized, their number is often underestimated by parents by a factor of 5 to 10.^15^

EEG findings also present reliability issues. The characteristic EEG pattern of IESS, hypsarrhythmia,^1^, ^16^ is only present in about 60% of cases^9^, ^10^ and its definition is unclear. The number of areas needed to qualify as multifocal and the amplitude at which the slow waves are considered large are undefined while chaoticity is intrinsically undefinable. In addition, several alternate types of modified hypsarrhythmia have been described.^9^, ^10^ Perhaps not surprisingly, a 2014 study by Hussain et al.^17^ showed poor interrater reliability (IRR) for diagnosing hypsarrhythmia and for most of its individual EEG components. Lack of definitions and validation has been an issue throughout history since the term hypsarrhythmia was first coined by Gibbs and Gibbs.^18^ Neither the 1984 article by Hrachovy et al.^19^ describing the various observed types of modified hypsarrhythmia nor the 2004 consensus statement of the West Delphi group concerning case definitions in IESS^20^ provide strict definitions of the phenomenon or its individual components. The three scoring patterns made by Bower and Jeavons,^21^ Rating et al.,^22^ and Kramer et al.^23^ were intended to meet the challenge of definitions, but they all lacked thorough measurements of IRR and none of them have subsequently been validated or widely used.

To provide clear definitions, Mytinger et al. developed the burden of amplitudes and epileptiform discharges (BASED) score in 2015.^24^ The score was revised in 2021^25^ to better account for phenomena like the slow waves of large amplitude commonly present in healthy infants^26^ and to include components of IESS not encompassed by the original iteration, such as paroxysmal voltage attenuation. The BASED score showed notably good IRR in the revisional study from 2021 and has started to be more widely used,^27^, ^28^, ^29^, ^30^, ^31^, ^32^ though it has yet to be thoroughly validated.

We hypothesized that the BASED score has higher IRR than the traditional method, while still being able to identify the epileptic encephalopathic nature of IESS. We aimed to evaluate interrater reliability of the BASED score in a Danish cohort with IESS and compare it with traditional hypsarrhythmia assessment.

## 2 METHODS

### 2.1 Procedures

We identified pre‐ and posttreatment EEGs from 20 consecutive patients with an initial EEG diagnosis of hypsarrhythmia between December 2020 and February 2024 from a local database, for which regional approval was obtained. Two EEGs were selected from each patient, one being the first EEG described with hypsarrhythmia and the other the first subsequent EEG without hypsarrhythmia. If the patient did not have an EEG recording without hypsarrhythmia, the latest EEG recording was used instead. At the time of the last EEG recording, the presence of clinical remission was determined from patient records, defined as remission of spasms. This was performed by one author (TSE), who was blinded to the subsequent EEG scores. The 5 min that visually had the most severe epileptic encephalopathic pattern were selected from each EEG for analysis (TSE); these typically occur during non‐REM sleep.

These 5‐min epochs, without identifying information, were then randomized and distributed to three experienced Danish neurophysiologists (BJ, EQ & VSH), who independently rated the EEGs. They first rated all EEGs according to the traditional approach^17^ and then using the 2021 BASED score,^25^ with the EEG order being different for each method. The raters were not allowed to retroactively change their designation of hypsarrhythmia after determining the BASED score. The three neurophysiologists received an introduction to the use of the BASED score which included the training video from the 2021 publication^25^ and a form for the individual components of the BASED score (Appendix S1). Raters were blinded to patient identity, clinical information, treatment status, and EEG order. Raters were given a deadline of a few months to complete the assessments.

When using BASED the raters evaluate the presence of seven individual components with a yes or no, these being: (1) normal EEG, (2) presence of interictal epileptiform discharges (simplified to “spikes”), (3) presence of multifocal spikes, (4) abnormally increased background amplitude, (5) presence of grouped multifocal spikes (GMFS), (6) electrodecrement (i.e., paroxysmal voltage attenuation), and (7) increased spike burden.^25^ Both the full longitudinal bipolar montage and a BASED montage for assessing background amplitude were available when scoring.

Since background amplitude should not be measured on a slow‐wave after a spike, it is not possible to reliably determine amplitude when the BASED score is 5. Therefore, EEGs where at least two of three raters assigned a BASED score of 5 were excluded from the sub analysis of abnormal high amplitude. Traditional assessment consisted of visual classification of hypsarrhythmia (present/absent), including modified hypsarrhythmia patterns, based on expert clinical interpretation.^18^ Consensus between raters was defined as the choice made by ≥2/3 of the raters in any dichotomous context.

The criteria for epileptic encephalopathy using the traditional method were any hypsarrhythmic or modified hypsarrhythmic pattern. When using the BASED score, the criteria were a score of 4 or higher, in accordance with the 2021 BASED score.^25^ Remission on EEG was defined as the disappearance of any hypsarrhythmia using the traditional method or as a decrease in the BASED score from ≥4 to ≤3, or from 3 to ≤2.^25^

### 2.2 Statistical analysis

IRR was calculated using Gwet's AC1^33^ to assess chance‐corrected agreement between the three raters when using the traditional method and when using the BASED score.

We used the same standard designations by Landis and Koch^34^ to determine the strength of the IRR as Mytinger et al. used^25^: 0.0–0.2 slight agreement, 0.21–0.40 fair agreement, 0.41–0.60 moderate agreement, 0.61–0.80 substantial agreement, and 0.81–1.0 near perfect agreement. Tests for independence on Gwet's AC1 were done by using AgreeTest®, an app developed by Gwet following the method for comparing both uncorrelated and correlated agreement coefficients with a 95% confidence interval as outlined by Gwet.^35^

Additionally, the number of EEGs with complete agreement between the raters was presented as a percentage. Comparisons of the complete agreement between groups were made using Fisher's exact test, as these were categorical variables from small groups. Paired nominal data like positive and negative predictive values, sensitivity and specificity were compared using McNemar's test. Statistical significance was defined as 5%.

All data analyses were done in Microsoft Excel and STATA.

No Artificial Intelligence Generated Content tools were used in any part of this article.

## 3 RESULTS

The median age of the patients at the first EEG recording was 9 months. Of the 20 patients, 7 were male and 13 were female. The mean time between the first and second EEG was 108 days (range 7–519). The etiologies varied, with none of the identifiable causes occurring more than twice; 6 out of 20 were unknown (Table 1). Death occurred in 3 out of 20 patients, but posttreatment EEGs were available for all three (Table 2).

**TABLE 1:** Patient characteristics.

	Sex (male/female)	Etiology	Age at 1st EEG (months)	Age at 2nd EEG (months)
Patient 01	Male	Unknown	7	8
Patient 02	Female	Sandhoff disease	15	16
Patient 03	Female	Pontocerebellar hypoplasia	24	44
Patient 04	Female	Congenital brain damage (CMV infection)	17	18
Patient 05	Male	Unknown	5	6
Patient 06	Female	Type 5 epileptic encephalopathy (PHACTR1)	5	6
Patient 07	Female	Unknown	4	5
Patient 08	Female	Cerebral palsy (Perinatal damage)	9	10
Patient 09	Female	Unknown	6	6
Patient 10	Female	Cerebral palsy (Neonatal asphyxia and sepsis)	6	6
Patient 11	Female	KCNQ2‐mutation	2	6
Patient 12	Male	Menkes syndrome	7	9
Patient 13	Female	Intrauterine infection	9	10
Patient 14	Male	Twin‐to‐twin transfusion syndrome and neonatal brain hemorrhage	7	14
Patient 15	Female	Neonatal meningitis (GBS infection)	21	38
Patient 16	Female	Pallister‐Kilian syndrome	51	56
Patient 17	Female	Pontocerebellar hypoplasia	25	29
Patient 18	Male	Unknown	18	18
Patient 19	Male	DNM1 encephalopathy	13	13
Patient 20	Male	Unknown	9	10

**TABLE 2:** Clinical and electrographic remission characteristics and consensus.

	Clinical remission	Fatal outcome	BASED score ≥4^a^ pre‐treatment ±	BASED score ≥4^a^ post‐treatment ±	Traditional HA^a^ pre‐treatment ±	Traditional HA^a^ post‐treatment ±
Patient 01	+	−	+	−	+	+
Patient 02	N/A^b^	+	+	+	+	−
Patient 03	−	−	+	+	+	+
Patient 04	+	−	+	−	+	−
Patient 05	+	−	+	−	+	−
Patient 06	+	−	+	−	+	−
Patient 07	+	−	+	−	+	−
Patient 08	+	−	+	−	+	−
Patient 09	+	−	+	−	+	−
Patient 10	+	−	+	−	+	−
Patient 11	+	−	+	−	−	−
Patient 12	−	+	+	+	+	+
Patient 13	+	−	+	−	+	−
Patient 14	−	−	+	+	+	+
Patient 15	−	+	+	+	+	+
Patient 16	+	−	+	−	+	−
Patient 17	−	−	+	+	+	+
Patient 18	+	−	+	−	+	−
Patient 19	+	−	+	+	+	+
Patient 20	+	−	+	−	+	−

Abbreviation: HA, hypsarrhythmia.

^a^ Based on consensus.

^b^ Unable to determine clinical remission due to hypotonic disorder.

All but one patient had spasms in relation to the initial EEG; this patient had a hypotonic disorder preventing the assessment of spasms (Sandhoffs disease, patient 2 in Tables 1 and 2). Of the remaining 19 patients, clinical remission was present in 14. Before treatment, there was consensus on epileptic encephalopathy for all these 19 patients using BASED (≥4) and on 18 out of 19 patients using the traditional method (any hypsarrhythmia). Post treatment, 13 out of 14 patients with clinical remission had remission on EEG using BASED (by consensus) and 12 out of 14 had remission using the traditional method (by consensus).

**FIGURE 1:**
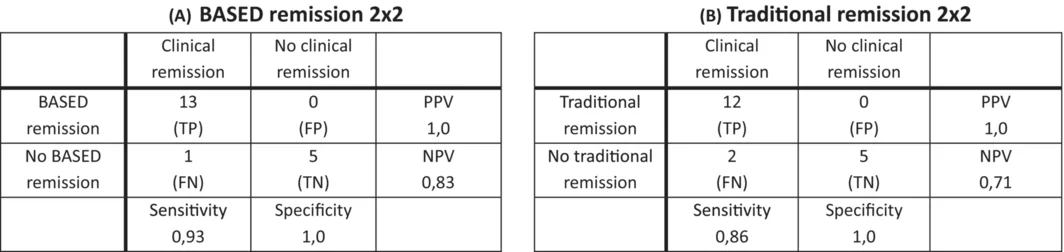
(A) BASED remission 2 × 2. (B) Traditional remission 2 × 2.

Both for the traditional method and for the BASED score method, there were no false positives for the evaluation of post‐treatment remission, meaning they never showed remission if the clinical signs showed a lack of remission (Figure 1A,B).

There was no difference between the two methods for positive predictive value, negative predictive value, specificity, nor sensitivity when comparing with McNemar's test (*p* = 1.0 in all cases).

Dichotomizing the BASED score into BASED ≥4 and BASED <4 there was a high IRR of 0.88 (95% CI: 0.76–1.00), with a complete agreement fraction of 36/40 for the EEGs. The IRR was near perfect or substantial in all but one (abnormal high amplitude) of the individual components of the BASED score (Table 3).

**TABLE 3:** Interrater reliability.

Component	Gwet's AC1	95% CI	Complete agreement
*Interrater reliability of the BASED score*			
BASED (exact score)	0.65 (S)	0.51–0.79	23/40 (57.5%)
BASED ≥4	0.88 (NP)	0.76–1.00	36/40 (90%)
Presence of spikes/sharpwaves	0.95 (NP)	0.87–1.00	38/40 (95%)
Presence of multifocal spikes/sharpwaves	0.85 (NP)	0.72–0.99	35/40 (87.5%)
Spikes in ≥50% of 1 s bins	0.70 (S)	0.52–0.88	31/40 (77.5%)
Abnormal high amplitude	0.54 (M)	0.20–0.88	12/19 (63%)
Grouped multifocal spikes	0.64 (S)	0.44–0.84	28/40 (70%)
Paroxysmal voltage attenuation	0.75 (S)	0.58–0.91	27/40 (67.5%)
*Interrater reliability of the traditional method*			
Hypsarrhythmia and/or modified hypsarrhythmia	0.62 (S)	0.42–0.81	28/40 (70%)
Hypsarrhythmia excluding modified hypsarrhythmia	0.54 (M)	0.33–0.75	26/40 (65%)
Modified hypsarrhythmia	0.40 (F)	0.14–0.65	19/40 (47.5%)

*Note*: SL: slight (0.00–0.20), F: fair (0.21–0.40), M: moderate (0.41–0.60), S: substantial (0.61–0.80), NP: near perfect (0.81–1.00).

Overall, the traditional method showed substantial agreement regarding hypsarrhythmia and modified hypsarrhythmia together (Table 3), with an IRR of 0.62 (95% CI: 0.42–0.81). The complete agreement fraction was 28/40 for all EEGs. For hypsarrhythmia without modified hypsarrhythmia, the IRR was 0.54 (95% CI: 0.33–0.75), and for modified hypsarrhythmia alone, it was 0.40 (95% CI: 0.14–0.65).

Agreement on the presence of an epileptic encephalopathy was better when using the quantitative criterion “BASED ≥ 4” than when using the qualitative traditional method, AgreeTest (*p* = 0.0104), and the proportion of patients with complete agreement between raters was also significantly higher for the BASED score (Fisher's exact test, *p* = 0.048, see Table 4). No other comparisons were significantly different.

**TABLE 4:** Comparison between the traditional method and the BASED score.

Any hyps AC1	BASED ≥4 AC1	*p*‐value, AC1	Any hyps CA	BASED ≥4 CA	*p*‐value, fisher
0.62 95% CI: 0.42–0.81	0.88 95% CI: 0.76–1.00	0.0104	28/40 (70%)	36/40 (90%)	0.048

Abbreviations: Any hyps, Hypsarrhythmia and/or modified hypsarrhythmia; CA, complete agreement.

## 4 DISCUSSION

We found a near‐perfect IRR using the BASED score to determine the presence of an epileptic encephalopathy (BASED ≥4), which was significantly better than the IRR using the traditional method. This finding is consistent with the findings of both the 2015 and the 2021 study by Mytinger et al.^24^, ^25^ It should be noted that our study found considerably better IRR when using the traditional method than Hussain et al.^17^ did in their studies critiquing the traditional method. Furthermore, the BASED scores of before and after treatment EEGs corresponded closely with the clinical course, supporting its validity for evaluating patients with IESS.

Mytinger et al.^24^, ^25^, ^26^ have made an important effort in introducing a quantitative method for the electrographic assessment of children with suspected IESS. The traditional visual method of assessment lacked strict definitions, which is likely why the BASED score shows better interrater reliability in multiple studies, including ours. It is noteworthy that the raters in the current study had at least 5 years of work experience together within the same center using the traditional hypsarrhythmia assessment and working as a closely knit team, including routinely reviewing EEGs together at consensus meetings. This background would be expected to favor high agreement for the traditional method, as we also found; however, there was an even higher agreement using the BASED score.

Several studies have been conducted using both the 2015 and 2021 BASED scores to evaluate treatment response and relapse risk, compare treatments, and to automate BASED scoring of EEG recordings.^27^, ^28^, ^29^, ^30^, ^31^, ^32^ A study protocol outlining a study of inter‐ and intrarater agreement when using BASED was published in 2022,^36^ but the subsequent study has yet to be produced. A 2025 study by Latzer et al.^37^ also found high interrater agreement using the BASED score to evaluate the interictal EEGs in both IESS and other developmental epileptic encephalopathies.^37^ However, that study used a different statistical approach—calculating the intraclass correlation coefficient (ICC) to measure interrater agreement rather than using Gwet's AC1 to measure interrater reliability, which was applied in the present study and the 2021 Mytinger et al. study.^25^ Latzer et al.^37^ also assessed interrater agreement for the Hypsarrhythmia Scoring System (HSS), another separate scoring system for hypsarrhythmia, when rating classical hypsarrhythmia and found moderate agreement. Notably, while the BASED scores were evaluated on 5‐min EEGs, HSS ratings were based on short 10‐s EEG segments which likely inflate the apparent agreement for the HSS score by reducing variability in the material rated.

Classically, some reservations have existed regarding the use of the BASED score to diagnose IESS or hypsarrhythmia, and Mytinger et al. emphasized that a BASED score ≥4 should not be equated directly with hypsarrhythmia.^25^ Interestingly, however, the findings by Latzer et al.^37^ suggest that a BASED score ≥4 may in fact discriminate IESS more specifically than classical hypsarrhythmia. In their cohort, 44% of patients with developmental and epileptic encephalopathies without spasms met the criterion BASED ≥4, compared with 55% who had an HSS ≥8, suggesting that hypsarrhythmia as defined by the HSS may be less specific for IESS than a high BASED score.

The limitations of this study include the relatively small sample size, which gives it less power to detect differences between groups. For example, the study was underpowered to compare positive and negative predictive values for remission. The design of selecting patients with pretreatment EEG diagnosis of hypsarrhythmia and a posttreatment EEG introduces potential bias, as patient selection may not fully represent the disease spectrum. Furthermore, the retrospective design using historical medical records is subject to variability in documentation quality, incomplete data, and lack of standardization in clinical assessments. The EEG samples used were the most abnormal 5 min to follow the original description of the BASED score, but in doing so one ignores large amounts of data in the EEG. As already noted, however, other studies on hypsarrhythmia may use only the most severe 10 s, which risks even larger bias toward better IRR.^37^ There was no structured interval between when the raters assessed the EEGs with each method, which could inflate the IRR by allowing the raters to recognize the EEGs the second time they saw them. None of the raters completed the ratings in one sitting, the sequence of EEGs was different when using the two methods, and the raters determined hypsarrhythmia first and BASED second, without the ability to amend the chosen hypsarrhythmia designation. A risk of bias toward a BASED score matching the hypsarrhythmia designation is still present and could have been lowered by designing an interval between the hypsarrhythmia scoring session and BASED scoring session.

In conclusion, this study showed that the 2021 BASED score is a tool with high IRR for the analysis of hypsarrhythmic patterns in EEGs from children with IESS. It also shows that the IRR using the BASED score is higher than for the traditional method. The BASED score could be used as a supplement to diagnose children with IESS in conjunction with the clinical picture. It might also be used to evaluate treatment response, but larger studies are needed to substantiate this.

## AUTHOR CONTRIBUTIONS

**T. P. Cramer:** Conceptualization, formal analysis, data curation, visualization, writing—original draft, project administration. **T. S. Engedal:** Conceptualization, methodology, investigation, data curation, project administration, supervision. **B. Johnsen:** Investigation, writing—review and editing. **V. S. Hansen:** Investigation, writing—review and editing. **E. Qerama:** Investigation, writing—review and editing. All authors critically revised the manuscript and approved the final version.

## FUNDING INFORMATION

This research received no external funding.

## CONFLICT OF INTEREST STATEMENT

None of the authors has any conflict of interest to disclose.

## PATIENT CONSENT

Informed consent was not required due to the retrospective nature of the study and the use of fully anonymized data, in accordance with national regulations.

**Test yourself**

1. What EEG threshold on the 2021 BASED score was used to define epileptic encephalopathy in this study, and how did its interrater reliability compare with the traditional hypsarrhythmia assessment?
2. Why was the BASED score developed, and what key limitation of traditional hypsarrhythmia assessment does it address?

*Answers may be found in the* *supporting information*.

## Supporting information


**Appendix S1** BASED scoring sheet.

Data S1.

## Data Availability

The data that support the findings of this study are not publicly available due to patient privacy and institutional restrictions, but they are available from the corresponding author upon reasonable request and with appropriate approvals.
