# Supplementary material for: Inter‐rater reliability and clinical utility of the BASED score in infantile epileptic spasms syndrome
Source: Epileptic Disord. 2026 Apr 21;28(4):1132–40. doi: 10.1002/epd2.70255 (PMC13499268; doi:10.1002/epd2.70255)
Supplement: Supplementary file 1 [file EPD2-28-1132-s001.docx]

**BASED score**

Review EEG in 15 second epochs.

**Question 1) Presence of multifocal spikes?**

Yes:	No:

Include irregular, "poorly formed" spikes – see stars in figure to the right.
Multifocal if ≥ 3 foci. May be present unilaterally.

**Question 2) Increased amplitude of the background?**

Yes:	No:

Channel: ___________	Measured amplitude: ________________

Measure in specific BASED score montage (see next page). Only count if recurring abnormal waves are present (at least one abnormal wave in at least 10/20 epochs).
Do not measure: Hypnagogic or hypnopompic rhythms or spike-and-waves.

(1)

**Do not measure waves as red arrows	Measure green waves**

**Question 3)**

**Grouped multifocal spikes?	Paroxysmal voltage attenuation?**

Yes:	No:	Yes:	No:

Grouped multifocal spikes (GMFS): At least 2 different spike foci in each hemisphere. When spikes are present in only one hemisphere, at least three different spike foci (which may include the midline) within a well-delineated group are required.

See the three arrows below (sensitivity 200 µV/cm):

**Question 4) Increased spike burden?**

Yes:	No:

All 15 second epochs are reviewed. For each 15 second epoch write the number of one second bins with spikes:

| Epoch | Number of bins | Epoch | Number of bins | Epoch | Number of bins |
| --- | --- | --- | --- | --- | --- |
| 1 |  | 8 |  | 15 |  |
| 2 |  | 9 |  | 16 |  |
| 3 |  | 10 |  | 17 |  |
| 4 |  | 11 |  | 18 |  |
| 5 |  | 12 |  | 19 |  |
| 6 |  | 13 |  | 20 |  |
| 7 |  | 14 |  |  |  |

Increased spike burden if at least 10/20 epochs have at least 8 one second bins with spikes.

**Background Amplitude Assessment Montage EEG-montage:**

References

1.	Mytinger JR, Vidaurre J, Moore-Clingenpeel M, Stanek JR, Albert DVF. A reliable interictal EEG grading scale for children with infantile spasms - The 2021 BASED score. Epilepsy Res. 2021;173:106631.
