# Supplementary material for: Inter‐rater reliability and clinical utility of the BASED score in infantile epileptic spasms syndrome
Source: Epileptic Disord. 2026 Apr 21;28(4):1132–40. doi: 10.1002/epd2.70255 (PMC13499268; doi:10.1002/epd2.70255)
Supplement: Supplementary file 2 [file EPD2-28-1132-s002.docx]

Answers

1. A BASED score ≥4 was used to define epileptic encephalopathy. It demonstrated near-perfect interrater reliability (Gwet's AC1 = 0.88), which was significantly higher than the traditional hypsarrhythmia assessment (AC1 = 0.62).
2. The BASED score was developed to provide clearer, quantitative definitions of EEG abnormalities in infantile epileptic spasms syndrome (IESS). Traditional hypsarrhythmia assessment lacks strict definitions (e.g., for “multifocal,” “high amplitude,” and “chaoticity”), leading to poor interrater reliability. The BASED score improves reliability by using defined, structured criteria.
